# Maternal Exposure of Rats to Isoflurane during Late Pregnancy Impairs Spatial Learning and Memory in the Offspring by Up-Regulating the Expression of Histone Deacetylase 2

**DOI:** 10.1371/journal.pone.0160826

**Published:** 2016-08-18

**Authors:** Foquan Luo, Yan Hu, Weilu Zhao, Zhiyi Zuo, Qi Yu, Zhiyi Liu, Jiamei Lin, Yunlin Feng, Binda Li, Liuqin Wu, Lin Xu

**Affiliations:** 1 Department of Anesthesiology, the First Affiliated Hospital, Nanchang University, Nanchang 33006, China; 2 Department of Anesthesiology, Jiangxi Province Traditional Chinese Medicine Hospital, Nanchang 33006, China; 3 Department of Anesthesiology, University of Virginia, Charlottesville, VA, 22908, United States of America; 4 Department of Anesthesiology, Jiangxi Province Tumor Hospital, Nanchang 330006, China; University of Pennsylvania, UNITED STATES

## Abstract

Increasing evidence indicates that most general anesthetics can harm developing neurons and induce cognitive dysfunction in a dose- and time-dependent manner. Histone deacetylase 2 (HDAC2) has been implicated in synaptic plasticity and learning and memory. Our previous results showed that maternal exposure to general anesthetics during late pregnancy impaired the offspring’s learning and memory, but the role of HDAC2 in it is not known yet. In the present study, pregnant rats were exposed to 1.5% isoflurane in 100% oxygen for 2, 4 or 8 hours or to 100% oxygen only for 8 hours on gestation day 18 (E18). The offspring born to each rat were randomly subdivided into 2 subgroups. Thirty days after birth, the Morris water maze (MWM) was used to assess learning and memory in the offspring. Two hours before each MWM trial, an HDAC inhibitor (SAHA) was given to the offspring in one subgroup, whereas a control solvent was given to those in the other subgroup. The results showed that maternal exposure to isoflurane impaired learning and memory of the offspring, impaired the structure of the hippocampus, increased HDAC2 mRNA and downregulated cyclic adenosine monophosphate (cAMP) response element binding protein (CREB) mRNA, N-methyl-D-aspartate receptor 2 subunit B (NR2B) mRNA and NR2B protein in the hippocampus. These changes were proportional to the duration of the maternal exposure to isoflurane and were reversed by SAHA. These results suggest that exposure to isoflurane during late pregnancy can damage the learning and memory of the offspring rats via the HDAC2-CREB -NR2B pathway. This effect can be reversed by HDAC2 inhibition.

## Introduction

Increasing evidence indicates that most general anesthetics are harmful to developing neurons and cause cognitive deficits in a dose- and time- dependent manner. Previous study [1] reported that exposure of pregnant rats to low concentrations of isoflurane (1.3%) for 6 hours did not cause neurodegeneration in the fetal brain or affect learning and memory in the offspring. However, in a similar animal model, exposure to high concentrations of isoflurane (3%) for only 1 hour caused significant neurodegeneration in fetal brain [2], suggesting a dose-dependent effect of isoflurane neurotoxicity. The majority of general anesthetics are lipophilic and can easily cross the placental barrier. About 0.5% to 2% of pregnant women will suffer non-obstetric surgery [3–5], and most of these procedures (up to 73%) must be completed under general anesthesia [6]. More than 75,000 pregnant women in the United States and 5,700 to 7,600 pregnant women in the European Union undergo non-obstetric surgery each year [7]. However, little is known regarding the effects of maternal exposure to general anesthetics during late pregnancy on the offspring’s subsequent learning and memory. Data from Sweden showed that among 5,405 patients who had non-obstetric surgery during pregnancy, 23% had procedures during the third trimester [4]. Most of the published studies about isoflurane showed a protective effect on the brain, however our previous studies showed that maternal exposure to propofol, ketamine, enflurane, isoflurane or sevoflurane during early gestation could cause learning and memory deficits and showed time-dependent effects [8]. A recent animal study indicated that rats exposed to isoflurane *in utero* at a time that corresponds to the second trimester in humans exhibited impaired spatial memory [9]. However, rats exposed to isoflurane on gestational day 21(E21) showed no neurotoxicity to the fetal brain, and no learning and memory impairments in the juvenile or adult rats [1].

Synaptic plasticity is critical to memory formation and storage [10]. Histone acetylation has been implicated in synaptic plasticity and learning and memory [11–13]. Histone deacetylase (HDAC) inhibitors can reinstate learning and promote the retrieval of long-term memory in animals with massive nerve degeneration [14]. These findings suggested that HDAC inhibition may provide a therapeutic avenue for memory impairment caused by neurodegenerative diseases. Among HDAC family members, HDAC2 functions in modulating synaptic plasticity and producing long-lasting changes to neural circuits, which in turn negatively regulate learning and memory [15]. The hyperphosphorylation of HDAC2 decreases the phosphorylation of cAMP response-element binding (CREB) protein, leading to a decrease in the CREB protein levels [16]. The administration of SAHA increased the levels of acetylated histones, accompanied by enhanced binding of phospho-CREB (p-CREB) to its binding site in the promoter of the NR2B gene, a subunit of N-methyl-D-aspartic (NMDA) receptors. This effect led to increased NR2B protein levels in the rat hippocampus, thus facilitating fear extinction [17]. Thus, HDAC2 modulates learning and memory by inhibiting CREB expression and down-regulating the expression of NR2B. Isoflurane can induce repression of contextual fear memory in 3-month-old mice by reducing histone acetylation in the hippocampus, an effect that can be rescued by the HDAC inhibitor sodium butyrate [18]. Neonatal mice repeatedly exposed to isoflurane also showed repression of contextual fear memory [19].

Many pregnancies include non-obstetric surgery during the late pregnancy due to diverse medical conditions, such as acute appendicitis, symptomatic cholelithiasis, and trauma [20–22]. Increasing reports suggested that any trimester of pregnancy should not be considered as a contraindication to surgery, and many non-obstetric surgeries can be safely performed in the third trimester [20–27]. Prospective clinical studies showed that approximately 27.6% of appendectomies performed during pregnancy were done in the third trimester, and none of the children exhibited any developmental delay during a 47.2-month (range from 13 to 117 months) follow-up time after delivery [28], however learning and memory was not evaluated in these children. The effect of maternal exposure to isoflurane on learning and memory and its mechanism is not well understood. Therefore, the present study was designed to explore the effects of maternal exposure of rats to isoflurane during late pregnancy (corresponding to the human third trimester) on learning and memory in the offspring. Further, we hypothesized that the detrimental effects of isoflurance on learning and memory are mediated through changes in the HDAC2-CREB-NR2B pathway, which we explored by administration of an SAHA.

## Experimental Procedures

### Subjects

This protocol was approved by the institutional review board of the First Affiliated Hospital of Nanchang University on the Use of Animals in Research and Teaching. Seventy-day-old female Sprague-Dawley (SD) rats (maternal rats) were supplied by the animal science research department of the Jiangxi Traditional Chinese Medicine College (JZDWNO: 2011–0030). The learning and memory functions of the parental rats were assessed with the MWM before mating. Female rats were then housed with a male rat (2 female: 1 male rat per cage) for mating. Pregnant rats were identified and divided into the isoflurane exposure 2h (I2), 4h (I4), 8h (I8) and control (C) groups (n = 10 per group) based on the MWM test results to minimize the effects of maternal differences in learning and memory.

### Anesthesia

On E18, gravid rats in the I2, I4 and I8 groups were exposed to 1.5% isoflurane (Abbott laboratories Ltd, Worcester, MA, USA) in 100% oxygen for 2, 4 and 8 hours, respectively, while those in the control group received 100% oxygen only. Electrocardiogram, saturation of pulse oximetry, and the respiratory rate of the rats as well as the inhaled concentration of isoflurane were monitored continuously with a Datex-Ohmeda ULT-I analyzer. The tail invasive blood pressure was monitored intermittently. The rectal temperature was maintained at 37 ± 0.5°C with heating pads. The exposure time began from the loss of the righting reflex. The depth and rate of breath was monitored. The exposure durations were selected because different lengths of surgeries are performed [29], and neuronal damage or apoptosis reaches a maximum when general anesthetic exposure time reaches 6 to 8 hours [30]. Our preliminary study showed that maternal exposure to 1.5% isoflurane for 8 hours did not significantly change blood pressure, blood glucose or venous blood gases. The concentration of isoflurane was selected because 1.5% isoflurane in 100% oxygen equals approximately 1 MAC (minimum alveolar concentration) in gestating rats and caused righting reflex loss in our preliminary studies. At the end of the exposure time, all of the rats were exposed to 100% oxygen for 30 min for anesthesia recovery in an anesthesia chamber (40 × 40 × 25 cm). If the cumulative time of SpO_2_ <95% and/or the systolic blood pressure (SBP) decreased by more than 20% of baseline more than 5 minutes, the dam would be excluded from the study, and another dam was selected to supplement the sample size, thereby excluding the harmful effect of maternal ischemia or hypoxia on offspring rats. Furthermore, to clarify whether exposure to isoflurane caused a significant effect on the internal environment of maternal rats, 10 additional rats at gestational day 18 were selected. Five were exposed to 1.5% isoflurane in 100% oxygen for 8 hours, and the other five were exposed to 100% oxygen for 8 hours. Femoral vein blood was harvested for blood gas analysis.

### Morris Water Maze (MWM) Test

The age of P30 in rat corresponds to preschool age in human [31]. Therefore, we evaluated the spatial learning and memory of the offspring begining on P30 with MWM according previous report [32]. All of the offspring were acclimated to the experimental environment for 30 min before testing. The Morris water maze is a black circular steel pool with a diameter of 150 cm and a height of 60 cm, filled with 24 ± 1°C water to a depth of 20 cm. A circular escape platform of 10 cm in diameter was submerged 1 cm below the water surface in the second quadrant. The swimming trail and speed of the rats was automatically recorded by the SLY-WMS Morris water maze test system (Beijing Sunny Instruments Co. Ltd., Beijing, China). The escape latency (time needed to find the platform), platform crossing times (number of times the rat swam across the submerged platform), and the target quadrant traveling time (time spent in the platform-hidden quadrant) were recorded automatically by the test system. The tests were begun at 9:00 am, one time per day for seven consecutive days. Each offspring rat was put into the pool to search for the platform one time per day for six days (training trial). The starting point was in the third quadrant, the farthest quadrant from the platform-hidden quadrant (the second quadrant in the present study, named the target quadrant). The rats were placed in the water facing the wall of the pool. The same starting point was used for each rat (with a colour marker on the pool wall). The animals were allowed to stay on the platform for 30 seconds when they found the platform. If an animal could not find the platform within 120 s, the escape latency was recorded as 120 s for that trial. The animal was then guided to the platform and allowed to stay on it for 30 s. On the seventh day, the platform was removed. Rats were allowed to swim for 120s to test their memory (platform-crossing times and target quadrant traveling time). The mean of the latencies, platform-crossing times and target quadrant traveling time of the offspring rats born by the same mother rat were calculated as the final results.

**Fig 1:**
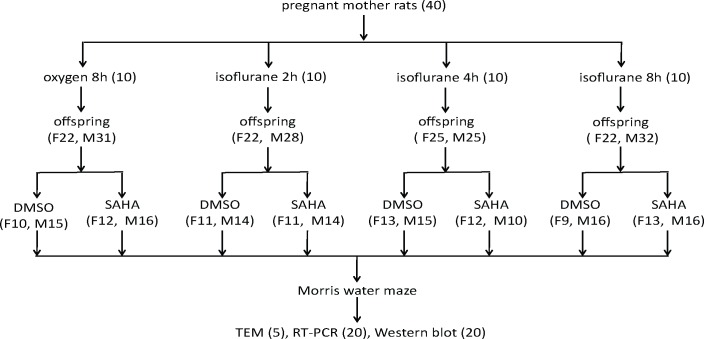
Experimental design. Pregnant dams were exposed to 1.5% isoflurane in 100% oxygen or to 100% oxygen alone for the times indicated on E18 and the offspring were treated with 90 mg/kg SAHA (ip) or vehicle (DMSO) 2 hours before behavioral testing. The number in parentheses represents the number of animals: F = female, M = male; DMSO = dimethyl sulfoxide; SAHA = suberanilohydroxamic acid, also known as vorinostat; TEM = transmission electron microscopy.

The offspring born to the same dam in each group were subdivided into the SAHA subgroup (I2S, I4S, I8S and CS subgroup) and the non-SAHA subgroup (I2N, I4N, I8N and CN subgroup) (Fig 1). Two hours before each MWM test, 90 mg/kg SAHA (Selleck Chemicals, Houston, TX, USA), at a concentration of 0.6 μM in dimethyl sulfoxide (DMSO) was given intraperitoneally to the offspring in the SAHA subgroups. An equal volume of DMSO was given to the rats in the non-SAHA subgroups. We selected 2 h before each MWM trial as the administration time point for SAHA based on the fact that 2 h after SAHA administration, the expression of NR2B increased in the hippocampus of Sprague-Dawley rats by enhancing histone acetylation, thus facilitating fear extinction [17].

### Transmission Electron Microscopy

The offspring were anesthetized with isoflurane at 24 h after the MWM test and then euthanized by cervical dislocation. Left hippocampus tissues were harvested quickly (in 1 minute) on ice and cut into small pieces of 1 mm^3^. The hippocampal pieces were immersed in 2.5% glutaraldehyde in 0.1 mol/l phosphate buffer (pH 7.4) at 4°C for 3 hours, rinsed three times in 0.1 M PBS (phosphate buffered saline), fixed in 1% osmium tetroxide at 4°C for 2 h, dehydrated, embedded, cut into ultrathin sections of 50–70 nm, stained by saturated uranium acetate and lead citrate and observed by transmission electron microscopy.

### Real Time Polymerase Chain Reaction (RT- PCR)

Total RNA of the hippocampus was extracted with Trizol reagent (Invitrogen) according to the manufacturer’s protocol. The mRNA concentration was measured (OD 260 nm) with a spectrophotometer (Nanophotometer P, MPLEN Co., Germany). Reverse transcription was performed with 1 μg total RNA using a Prime Script^TM^ RT reagent Kit with gDNA Eraser (Perfect Real Time; RR047A, TaKaRa BIO Inc., Japan). The cDNA sample was amplified by a real time PCR instrument (ABI7500), with SYBR Premix Ex Taq^TM^ (Tli RNaseH Plus; Code: RR820A, TaKaRa Co., Japan). β-actin was chosen as a reference gene. The length of both the HDAC2 product and the CREB product is 94 bp, whereas the length of the NR2B product is 103 bp, and the length of the β-actin product is 150 bp. PCR amplification was performed with the following cycling parameters: one cycle of 95°C for 30 s followed by 40 cycles of 95°C for 5 s, 60°C for 34 s, 95°C for 15 s, 60°C for 1 min and 95°C for 15 s.

The ABI7500 instrument automatically analyzed the fluorescence signal and converted it to the Ct value, using β-actin as a housekeeping gene and the Ct value of group C as the comparative object. Single-product amplification was confirmed by melting curve and gel electrophoresis analysis. The expression levels of HDAC2, CREB and NR2B mRNA were normalized to β-actin mRNA and the values of the control group. The mean mRNA expression level of all of the offspring born to the same mother rat was calculated as the final expression level of mRNA.

### Western Blot

Total protein was extracted by lysing the hippocampus (one offspring from each dam) in lysis buffer (Thermo Scientific, Rockford, IL, USA) containing a protease inhibitors cocktail (Sigma-Aldrich). Total protein (50 μg/ lane) was separated on a polyacrylamide gel and then transferred onto PVDF membranes. The membranes were blocked with Protein-Free T20 Blocking Buffer (Thermo Scientific) for 1 h at room temperature and incubated with rabbit polyclonal anti-NR2B antibody (Cell signaling Technology, 1:500) or rabbit polyclonal anti-β actin antibody (Cell Signaling Technology, 1:500) overnight at 4°C. After incubation with goat anti-rabbit HRP-conjugated IgG, the protein complex was revealed with enhanced chemiluminescence reagents (Pierce, IL, USA) and quantified by Genesnap version 7.08. The density of NR2B protein band was normalized to that of β-actin in the same sample. The results from isoflurane exposed offspring were then normalized to the average values of control offpring in the same western blot. The mean expression level of all of the offspring born to the same mother rat was calculated as the final expression level of NR2B protein.

### Statistical Analysis

All values shown represent the mean ± SEM. The escape latency was subjected to a two-way repeated measures ANOVA (RM ANOVA) with prenatal treatment as a between-litters independent factor and day as a repeated factor. When an initial ANOVA showed main effects of the factors as well as significant interactions among the factors, post hoc comparisons were conducted by the least significant difference (LSD) *t* test. The mRNA and protein data, platform crossing times, and target quadrant traveling time were analyzed by one-way ANOVA, and followed by LSD *t* test when a significant difference was found in groups (*p* < 0.05). Results are considered statistically significant at *p* < 0.05.

## Results

### Isoflurane Exposure Does Not Alter Maternal Blood Gases

To clarify whether exposure to 1.5% isoflurane for 8 hours causes significant changes to the internal environment during late pregnancy, 10 gravid rats on E18 were used. We continuously monitored the saturation of pulse oximetry during anesthesia. As a matter of convenience, femoral vein blood gas analysis was used to evaluate whether isoflurane exposure would cause changes in acid-base balance or serum electrolytes in maternal rats. All of the indices of venous blood gases showed no significant changes after an 8-hour exposure to 1.5% isoflurane compared with rats exposed to oxygen only (Table 1). These results indicate that exposure to 1.5% isoflurane for 8 hours on E18 does not cause significant metabolic changes to pregnant rats.

**Table 1:** The effect of isoflurane exposure on femoral venous blood gas and electrolytes in maternal rats.

Indexes	100% oxygen	1.5% isoflurane+100% oxygen
pH	7.39±0.03	7.39±0.23
PO_2_ (mmHg)	46.33±6.15	49.50±4.93
PCO_2_ (mmHg)	51.5±3.62	50.50±12.79
HCO_3_^-^ (mmol/L)	31.13±0.45	27.85±4.78
BE(B) (mmol/L)	3.75±1.33	3.20±0.80
Ca^2+^ (mmol/L)	1.24±0.20	1.41±0.06
K^+^ (mmol/L)	4.51±0.64	4.8±0.50
Na^+^ (mmol/L)	135.50±1.22	134.25±0.96

Rats were exposed to oxygen or isoflurane + oxygen for 8 hour and monitored continuously. Final values were recorded at the end of the 8 hour period. n = 5.

### Impaired Learning and Memory in Rat Offspring and the Ameliorating Effect of SAHA

**Fig 2:**
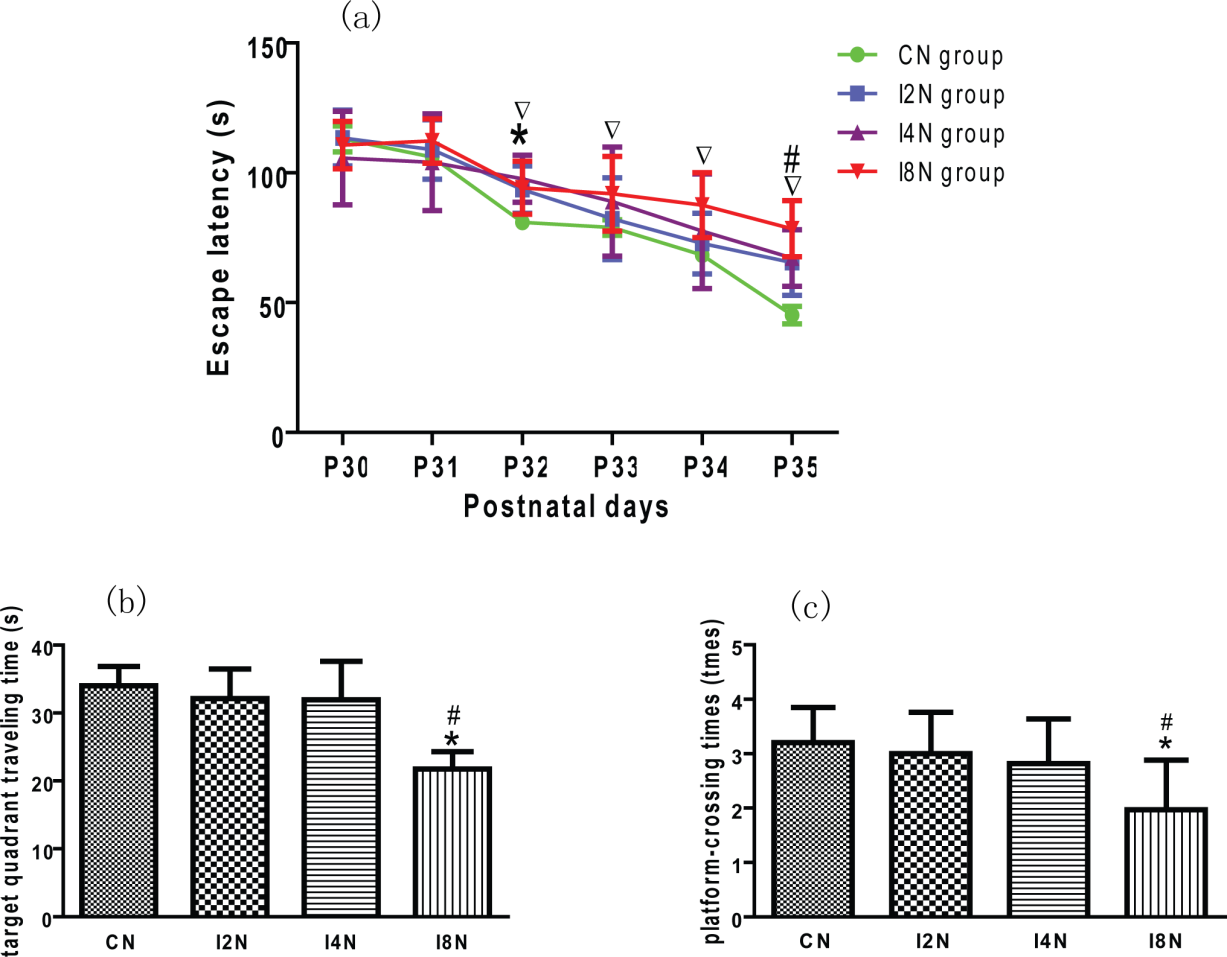
Maternal isoflurane exposure impaired learning and memory in offspring: Offspring of rats exposed to isoflurane on gestation day 18 (E18) for 2h (I2N), 4h (I4N) and 8h (I8N) respectively. Thirty days postneaonatal (P30), the learning and memory was assessed using the Morris water maze: (a) Escape latency (time to find the hidden platform). At the third trial, the escape latency in the I2N, I4N or I8N group was significant longer than the control group (^*^ p < 0.05); The escape latency increased with the increase of isoflurane exposure time. The escape latency in I8N group was significant longer than control group at 3^rd^, 4^th^, 5^th^ and 6^th^ trial (^▽^ p < 0.05). At the 6^th^ training trial, the escape latency in the I8N group was significantly longer than the I2N or I4N group (^#^ p < 0.05); (b) Target quadrant traveling time. The offspring in isoflurane exposure group spent less time traveling in the platform hidden quadrant (target quadrant). The target quadrant traveling time in I8N group was significant less than CN group (^*^ p < 0.05), I2N and I4N group (^#^ p < 0.05); (c) Platform crossing times. The offspring in isoflurane exposure group swam across the location where the platform hidden (platform-crossing times) less than control group, especially those in I8N group. The platform-crossing times in I8N group was significant less than CN group (^*^ p < 0.05), I2N and I4N group (^#^ p < 0.05). CN = control group.

The results of MWM showed that the offspring in isoflurane exposure group had to spend more time finding the platform than the control group. At the third MWM trial, the escape latency in the I2N, I4N or I8N groups was longer than the control group (*p* < 0.05). The escape latency increased with the increase of isoflurane exposure time. The escape latency in I8N group was longer than control group at 3^rd^, 4^th^, 5^th^ and 6^th^ trial (^▽^ p < 0.05). At the 6^th^ training trial, the escape latency in the I8N group was significantly longer than the I2N or I4N group (^#^ p < 0.05) (Fig 2A). The offspring in isoflurane exposure group spent less time traveling in the platform hidden quadrant. The target quadrant traveling time in I8N group was less than CN group (* p < 0.05), I2N and I4N group (^#^ p < 0.05). The offspring in isoflurane exposure group swam across the location where the platform hidden less than control group, especially those in I8N group. The platform-crossing times in I8N group was less than CN group (* p < 0.05), I2N and I4N group (^#^ p < 0.05, Fig 2B and 2C). These results indicate that maternal exposure to isoflurane of approximately 1 MAC (1 MAC of isoflurane for rats on gestational day 14–16 is 1.4%) [33, 34] can impair learning and memory in the offspring.

**Fig 3:**
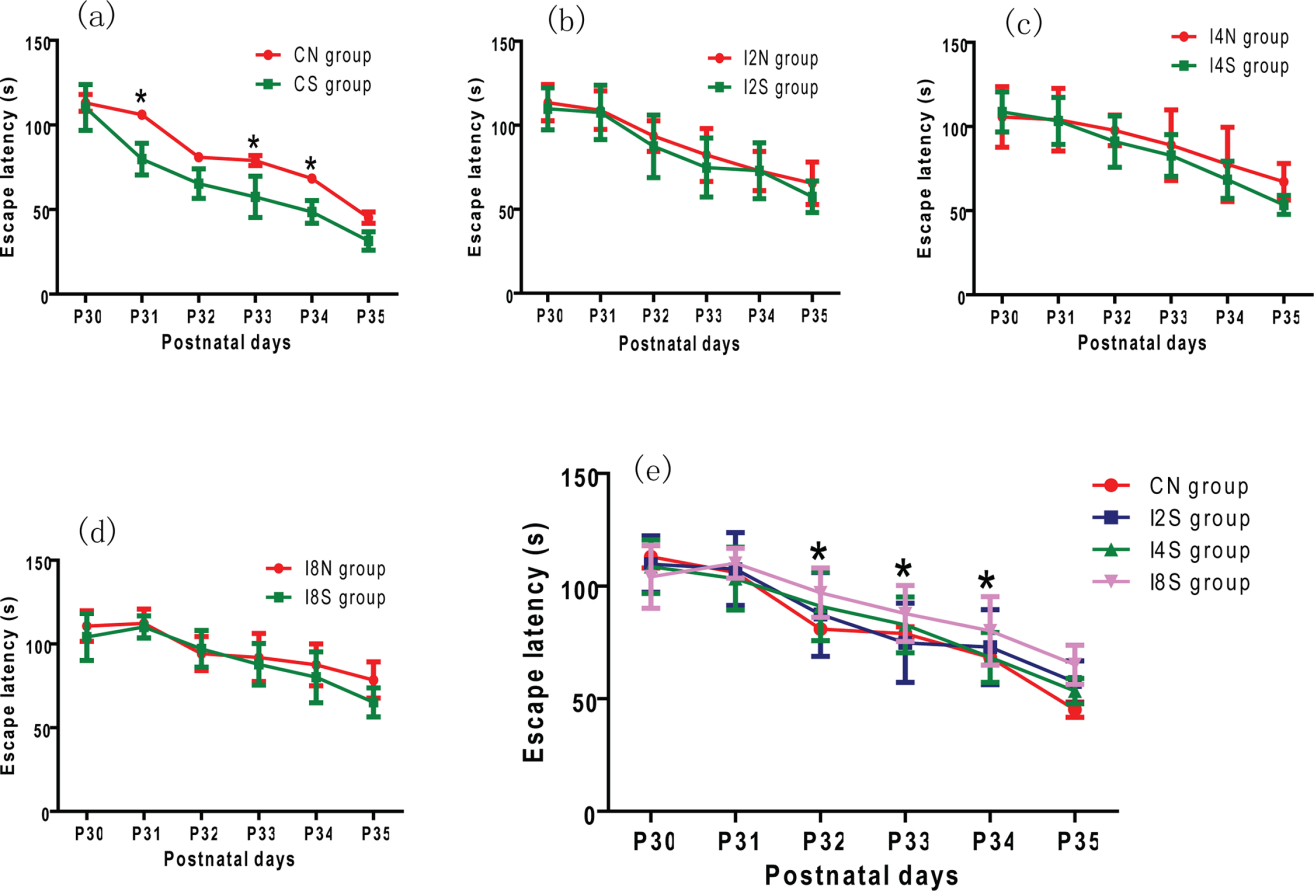
HDAC2 inhibition alleviated the impaired learning caused by maternal isoflurane exposure. SAHA potentiates the learning ability of normal rats, the escape latencey in SAHA treated normal offspring was shorter than normal control offspring at 2^nd^, 4^th^ and 5^th^ trial (* p < 0.05, Fig 3a); The escape latency in I2S, I4S and I8S group were shorter than their relative control groups (I2N, I4N and I8N group respectivly), but had no statistical differences (p > 0.05, Fig 3b, c and d). The escape latency in I8S group was longer than normal control group (CN group) at 3^rd^, 4^th^ and 5^th^ trial (p < 0.05, Fig 3e). S = SAHA treated subgroup; N = non—SAHA treated subgroup.

**Fig 4:**
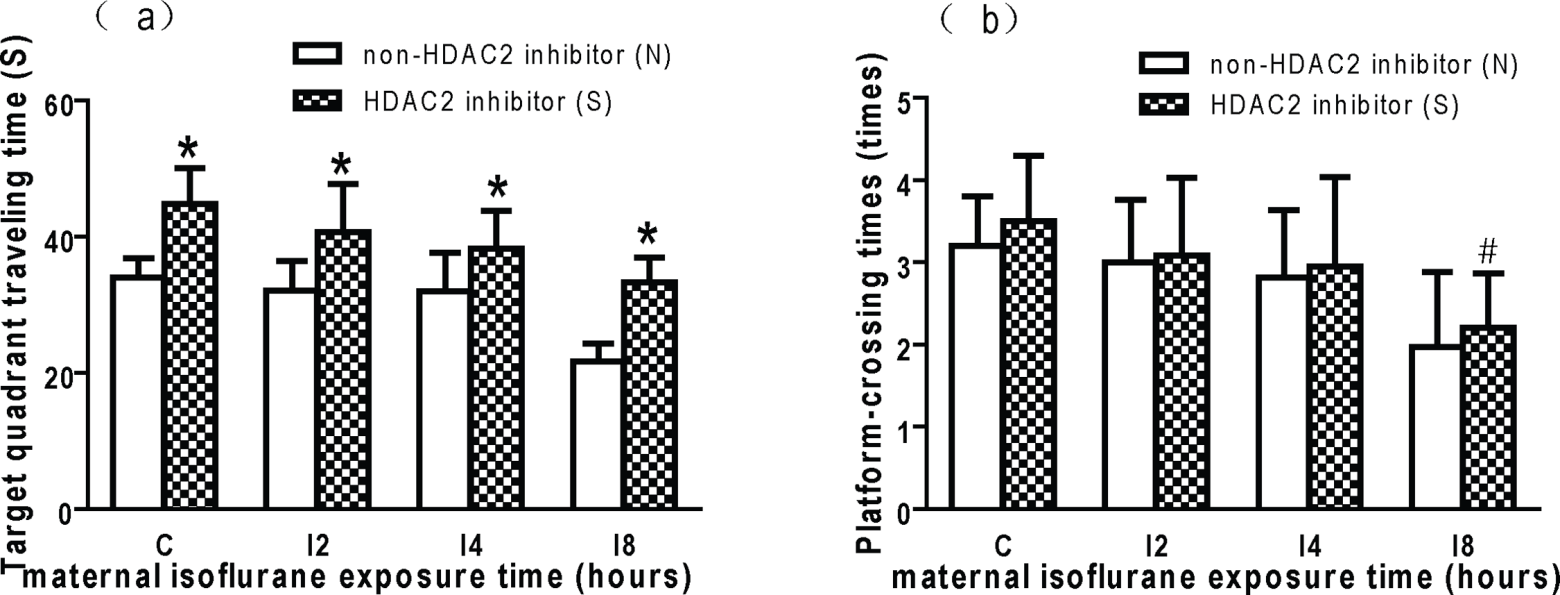
HDAC2 inhibition reversed the memory impairment caused by maternal isoflurane exposure. (a) Target quadrant traveling time. The target quadrant traveling time in SAHA treated sugroup was significant more than relative non-SAHA subgroups (* p < 0.05); The traveling time in I2S, I4S and I8S group was not significantly different from that in CN group. (b) Platform crossing times. The platform-crossing times in SAHA treated sugroups increased compared with their relative non-SAHA subgroups, but had no statistical differences. The platform-crossing times in I8S subgroup was still less than CN group (* p < 0.05).

To study whether the learning and memory impairment caused by maternal isoflurane exposure could be reversed by HDAC inhibitor, SAHA was given to the offspring before each MWM trial. The escape latencey in SAHA treated normal offspring was shorter than control group at 2^nd^, 4^th^ and 5^th^ trial (* p < 0.05, Fig 3A). The escape latency in I2S, I4S and I8S group were shorter than their relative control groups (I2N, I4N and I8N group respectivly), but had no statistical differences (p > 0.05, Fig 3B, 3C and 3D). The escape latency in I8S group was longer than normal control group (CN group) at 3^rd^, 4^th^ and 5^th^ trial (p < 0.05, Fig 3E). The target quadrant traveling time in SAHA treated sugroup was more than relative non-SAHA subgroup (p < 0.05, Fig 4A). The traveling time in I2S, I4S and I8S group was not significantly different from that in CN group (Fig 4A). The platform-crossing times in SAHA treated sugroups increased compared with their relative non-SAHA subgroups (Fig 4B). But the platform-crossing times in I8S subgroup was still less than CN group (p < 0.05, Fig 4B). These results indicate that SAHA can alleviate the learning and memory impairment caused by isoflurane exposure, but cannot completely reverse the impairment when the exposure time was 8 hours.

### Maternal Isoflurane Exposure Disrupted Ultrastructural Features of Hippocampal Neurons in Offspring

**Fig 5:**
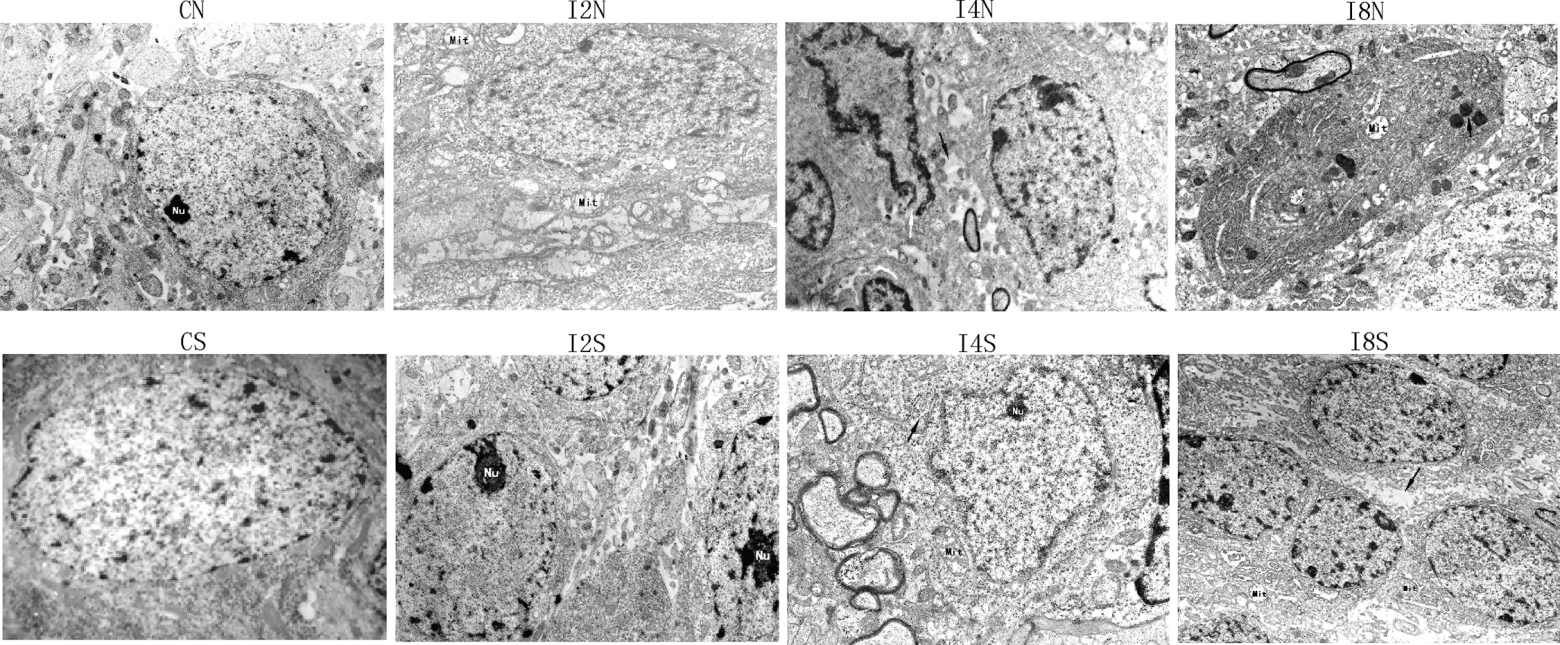
HDAC2 inhibition alleviated the hippocampal ultrastructure impairment caused by maternal isoflurane exposure (transmission electron microscopy, ×6000). The hippocampal ultrastructure showed apparent abnormality with the increase of isoflurane exposure time. The ultrastructure showed no differences compared to the control group when isoflurane exposure time was 2h (I2N). When isoflurane exposure time lengthen to 4h, the neuron number decreased, the nuclei became irregular, cytoplasmic area decreased, mitochondrial number decreased, and we observed evidence of disordered mitochondrial cristae. The quantity of rough endoplasmic reticulum, ribosome and Golgi apparatus decreased, and the ribosomes exhibited degranulation (I4N). When the exposure time prolonged to 8h, all of these changes became more prominent, there were fewer neurons with dilated intercellular space. Dissolved mitochondrial cristae and swollen Golgi apparatus could be observed (I8N). HDAC inhibition alleviated the impairments, but did not increase the neuronal number (I4S and I8S group).

Ultrastructural changes in hippocampal neurons were evaluated by electron microscopy. Maternal isoflurane exposure impaired the structure of the hippocampus when the exposure time was more than 4 hours. The ultrastructure in group I2N showed no obvious differences compared to group CN. When the exposure time was lengthened to 4 hours, neuron number decreased, nuclei became irregular, cytoplasmic area decreased, mitochondrial number decreased, and we observed evidence of disordered mitochondrial cristae. The quantity of rough endoplasmic reticulum, ribosome and Golgi apparatus decreased, and the ribosomes exhibited degranulation. When the isoflurane exposure time was prolonged to 8 hours, all of these changes became more prominent. We observed fewer neurons with dilated intercellular space. Dissolved mitochondrial cristae and swollen Golgi apparatus could be observed in group I8. HDAC inhibition alleviated all the hippocampal impairments caused by isoflurane exposure, but the neurons number had no obvious change (Fig 5).

### Increased HDAC2 mRNA Expression Caused By Isoflurane and the Reversed Effect of SAHA

**Fig 6:**
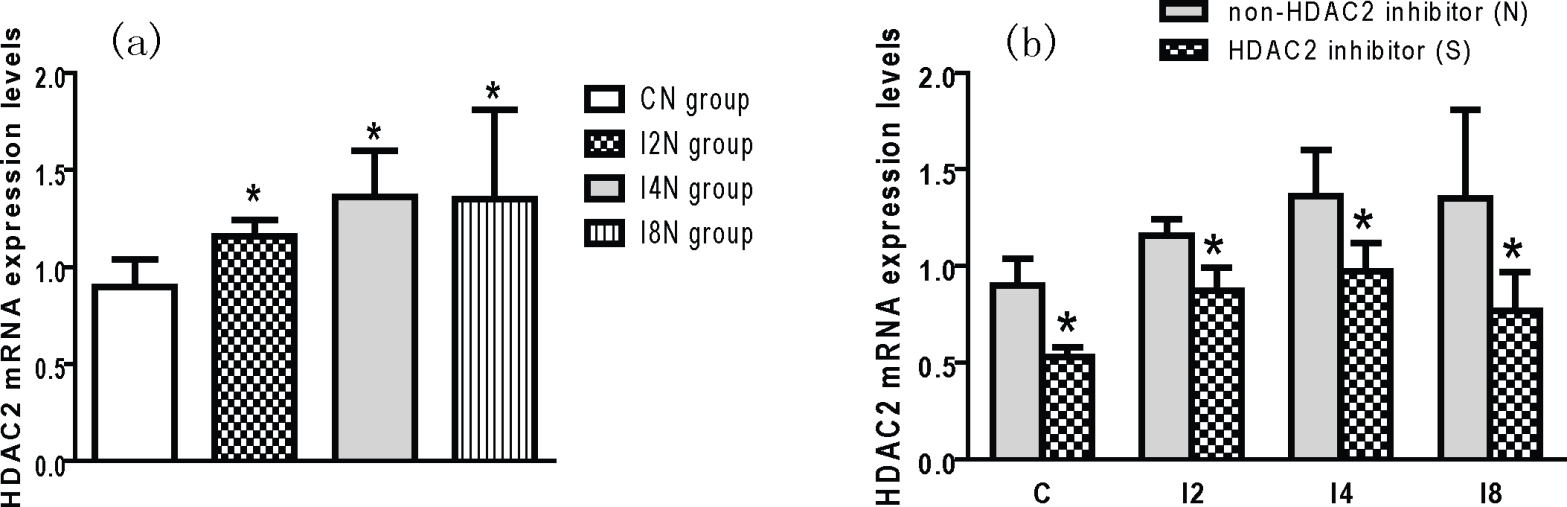
HDAC2 inhibition reversed the overexpression of HDAC2 mRNA caused by maternal isoflurane exposure. The expression levels of HDAC2 mRNA in offspring hippocampus were detected by real time PCR (RT—PCR). The levels of mRNA were normalized to that of β-actin in the same sample and then normalized to the average values of control offspring in the same RT-PCR. The mean value of the mRNA expression level of all of the offspring born to the same mother rat was calculated as the final expression level of mRNA. (a) maternal isoflurane exposure potentiated the expression of HDAC2 mRNA. The HDAC2 mRNA levels in the offpsring hippocampus in I2N, I4N and I8N group were higher than normal control group (CN group, * p < 0.05). (b) SAHA reversed the overexpression of HDAC2 mRNA. The HDAC2 mRNA levels in SAHA treated subgroup were higher than non-SAHA subgroups (* p < 0.05).

Maternal isoflurnae exposure increased the expression levels of HDAC2 mRNA in the hippocampus of the offspring rats (*p* < 0.05; Fig 6A). SAHA reversed the expression of HDAC2 mRNA in the hippocampus. The expression levels of HDAC2 mRNA in the CS, I2S, I4S and I8S groups were lower than those in the CN, I2N, I4N and I8N groups respectively (*p* < 0.05; Fig 6B). These results indicate that HDAC2 inhibition can reverse the overexpression of HDAC2 mRNA in offspring caused by maternal isoflurane exposure during late pregnancy.

### Downregulated CREB mRNA Expression and the Reversed Effect of SAHA

**Fig 7:**
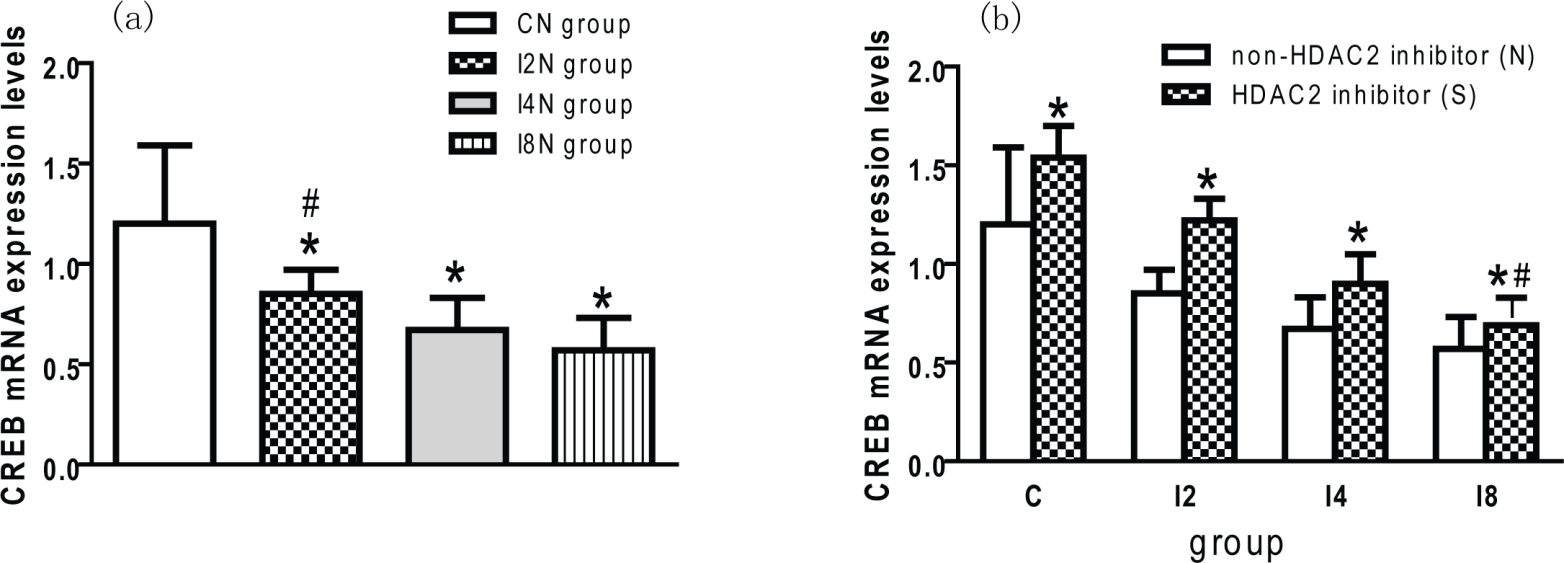
HDAC2 inhibition reversed the downregulated expression of CREB mRNA caused by maternal isoflurane exposure: (a) Isoflurane exposure downregulated CREB mRNA expression. The CREB mRNA levels in the offspring hippocampus in isoflurane exposed group were lower than control group (* p < 0.05). With the increase of isoflurane exposure time, the downregulated effect became more obvious. The CREB mRNA levels in I8N group were higher than I2N group (^#^ p < 0.05). (b) SAHA reversed the down-regulation of CREB mRNA expression. Compared with relative non-SAHA subgroup, the levels of CREB mRNA in SAHA treated sugroup increased (* p < 0.05). But the CREB mRNA levels in I8S subgroup were still lower than normal control group (^#^ p < 0.05). This indicates that SAHA cannot completely reverse the downregulated effect caused by isoflurane when the exposure time prolonged to 8 hours.

The expression levels of CREB mRNA in the hippocampus of the offspring in the I2N, I4N and I8N groups were significantly lower than those in the CN group (*p* < 0.05; Fig 7A). These results indicate that maternal isoflurane exposure during late pregnancy can inhibit the expression of CREB mRNA in offspring. The CREB mRNA levels in the I8N group were significantly lower than in the I2N group and I4N group (*p* < 0.05; Fig 7A), suggesting that prolonged exposure to isoflurane during late pregnancy has a more profound effect on inhibiting CREB mRNA expression. The expression levels of CREB mRNA in the CS group were higher than those in the CN group (*p* < 0.05; Fig 7B). This finding indicates that SAHA can potentiate the expression of CREB mRNA in the hippocampus of the offspring rats. The expression levels of CREB mRNA in the I2S, I4S and I8S groups were significantly higher than those in the I2N, I4N and I8N groups respectively (*p* < 0.05; Fig 7B). These results indicate that SAHA can reverse the inhibiting effect of maternal isoflurane exposure on CREB mRNA expression offspring rats. However, the expression level of CREB mRNA in the I8S group was still lower than CN group (p < 0.05, Fig 7B). This means that SAHA cannot completely reverse the inhibiting effect of maternal isoflurane exposure on the expression of CREB mRNA when the exposure time is 8 hours.

### Downregulated Expression of NR2B and the Reversed Effect of SAHA

**Fig 8:**
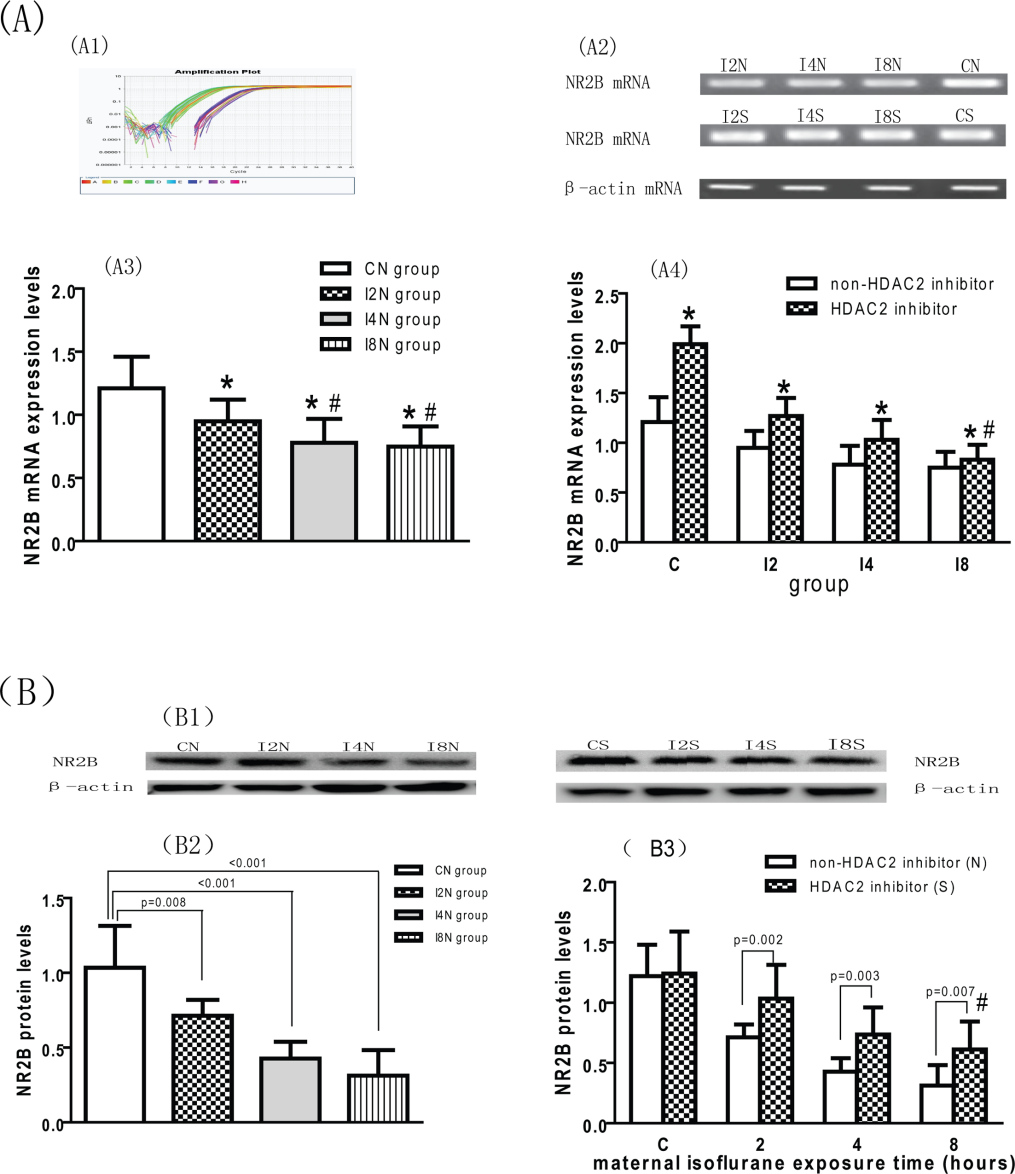
HDAC2 inhibition reversed the downregulated expression of NR2B caused by maternal isoflurane exposure. (A1) Amplification plot of NR2B and β-actin mRNA; (A2) Agarose gel electrophoresis images of NR2B and β-actin mRNA; (A3) Maternal isoflurane exposure downregulated the expression of NR2B mRNA (mean ± SE): The levels of NR2B mRNA in isoflurane exposure group (I2N, I4N, I8N) were lower than CN group (* p < 0.05). The NR2B mRNA levels in I4N and I8N group were lower than I2N group (^#^ p < 0.05); (A4) SAHA reversed the downregulated expression of NR2B mRNA (mean ± SE): The levels of NR2B mRNA in I2S, I4S and I8S subgroup were higher than I2N, I4N and I8N subgroup respectively (* p < 0.05). The NR2B mRNA levels in I8S subgroup were lower than CN group (^#^ p < 0.05). (B1) NR2B protein western blot images; (B2) Maternal isoflurane exposure downregulated the expression of NR2B protein (mean ± SD): The protein levels of NR2B protein were lower than CN group (^#^ compared with I2N group, p<0.05b); (B3) HDAC2 inhibition reversed the downregulated expression of NR2B protein: The protein levels of NR2B in I2S, I4S and I8S subgroups were higher than I2N, I4N and I8N sugroups respectively. The levels of NR2B protein in I8S subgroup were lower than CN group (^#^ p < 0.001). This indicates that SAHA cannot completely reverse the downregulated effect caused by isoflurane when the exposure time prolonged to 8 hours.

The expression levels of NR2B mRNA in the hippocampus of the offspring in the I2N, I4N and I8N groups were lower than those in the CN group (*p* < 0.05; Fig 8A3). The expression levels of NR2B mRNA in the I4N and I8N groups were lower than those in the I2N group (*p* <0.05; Fig 8A3). The changes of NR2B protein expression levels were similar to NR2B mRNA levels (Fig 8B3). These results indicate that maternal isoflurane exposure during late pregnancy inhibits the expression of NR2B in the hippocampus of the offspring rats and that prolonged isoflurane exposure can exacerbate these changes. The expression levels of NR2B protein and mRNA in the CS, I2S, I4S and I8S groups were higher than those in the CN, I2N, I4N and I8N groups respectively (Fig 8A4 and 8B3). These results indicate that SAHA can reverse the inhibiting effect of maternal isoflurane exposure on NR2B expression in the hippocampus of the offspring. However, the mRNA and protein levels of NR2B in the I8S group were still lower than those in the CN group (Fig 8A4 and 8B3). Thus, HDAC2 inhibition cannot completely reverse the inhibiting effects of maternal isoflurane exposure on the expression of NR2B when the exposure time is 8 hours.

## Discussion

The present study provides preclinical evidence that maternal rat exposure to isoflurane during late pregnancy impairs the spatial learning and memory in their offspring. The behavioural abnormality was associated with hippocampal neuronal damage, overexpression of HDAC2 mRNA and the subsequent downregulation of CREB mRNA and NR2B in hippocampus.

This finding is similar to the results described in a previous report, which showed that maternal exposure to 1.4% isoflurane for 4 hours on gestational day 14 impairs the offspring rats’ spatial memory acquisition [9]. The results of the present study are different from another report showing that isoflurane exposure during late pregnancy was not neurotoxic to the fetal brain and did not impair learning and memory in juvenile or adult offspring [1]. Isoflurane neurotoxicity is concentration-dependent [35]. General anesthetics can be neuroprotective and neurotoxic, depending on the levels, timing and mode of exposure. Isoflurane exerts multiple effects on neuronal stem cell survival, proliferation and differentiation. Short exposures to low isoflurane concentrations promote proliferation and differentiation of ReNcell CX cells, whereas prolonged exposure to high isoflurane concentrations induced significant cell damage [36]. This may be one of the critical reasons that our result is different from the report by Li et al. [1]. Our study involved 1.5% isoflurane, approximately 1 MAC, which is higher than the concentration used in the previous study (1.3%) [1]. The different exposure timing may be another reason for the differences between two experiments. The exposure time point in our experiment was E18 day, whereas that in previous study was E21 [1]. Therefore, the developmental maturity of the neurons was different between two experiments, which may result in different vulnerability to isoflurane [37]. In the present study, the dams were divided into different groups based on their learning and memory results tested before pregnancy. This was meant to exclude the effects of genetic factors on the learning and memory of the offspring and may have facilitated the significant differences in learning and memory obtained between the control and isoflurane exposure groups.

There was no obvious dyskinesia in offspring. There was no difference in swimming speed (automatically record by MWM system) of the offspring among groups. We had not evaluated the anxiety of offspring rats in present study. But previous study had revealed that rats exposed to isoflurane in utero on E14 have reduced anxiety compared with controls [9]. Thus the differences in learning and memory results were not caused by abnormal motor function or anxiety in offspring. The femoral vein blood gas analysis showed that exposure to this concentration of isoflurane for 8 hours had no significant effect on the blood gases and electrolytes of the dams (Table 1). Normal maternal body temperature was maintained during the isoflurane exposure process. Dams were removed from the study if they exhibited a cumulative time of more than 5 minutes with SpO_2_ <95% or >20% decrease in systolic blood pressure (SBP). Therefore, the damaging effect on learning and memory was not induced by physiological disturbances caused by isoflurane.

How does isoflurane exposure damage the spatial learning and memory function in offspring? Transmission electron microscopy results showed that neuron number and ultrastructure in offspring hippocampus had been impaired (Fig 5). HDAC inhibitors could alleviate the impairments, thus improving learning and memory. These data suggest that maternal exposure to isoflurane during late pregnancy harms hippocampal neurons, thus impairing learning and memory in the offspring. Previous results showed that prenatal exposure to 1.3% isoflurane for 4 hours on gestational day 14 led to impaired synaptic ultrastructure in the hippocampus of the offspring and thus causing poor learning and memory [38].

Recently, histone acetylation, which is regulated by histone deacetylases (HDAC), has been implicated in memory formation [39–43]. Increasing histone-tail acetylation can facilitate learning and memory [12, 13]. Further studies showed that HDAC2, but not HDAC1, decreases dendritic spine density, synapse number, synaptic plasticity and memory formation [15]. HDAC2 regulates learning and memory via the transcription factor CREB and the recruitment of the transcriptional coactivator and histone acetyltransferase CREB-binding protein (CBP) via the CREB-binding domain of CBP [44]. The inhibition of HDAC can modulate hippocampal-dependent long-term memory in a CBP-dependent manner [45]. Inhibiting HDAC increases the levels of acetylated histones and phospho-CREB (p-CREB), which enhances the binding of p-CREB to its binding site at the promoter of the NR2B gene, thus increasing the expression of NR2B in the hippocampus [17]. Thus, HDAC2 impairs learning and memory through a pathway involving HDAC2-CREB-NR2B. NR2B is critical to the formation and maintenance of learning and memory [46–48]. It is undetermined whether maternal isoflurane exposure during late pregnancy damage the spatial learning and memory in offspring via this pathway. The hippocampus is a critical structure for learning and memory. Thus, the expression levels of HDAC2, CREB, and NR2B mRNA and NR2B protein in the hippocampus of the offspring were analyzed in the present study. The results showed that maternal isoflurane exposure during late pregnancy increased the expression of HDAC2 mRNA, decreased CREB mRNA and NR2B mRNA and protein in the hippocampus of the offspring. This is similar to a previous report that maternal anesthesia with ketamine on G14 downregulated the expression of NR2B in the hippocampus of offspring [49]. These results indicate that maternal isoflurane exposure during late pregnancy causes the over-expression of HDAC2, thereby inhibiting the expression of CREB mRNA, resulting in downregulation of NR2B in the hippocampus of the offspring rats. These effects lead to impaired learning and memory in the offspring. NMDA receptor blockade acts critical role in determining whether neurons are reversibly injured or are driven to cell death by isoflurane [50]. Thus, these results indicate that maternal isoflurane exposure during late pregnancy can damage the learning and memory of the offspring rats via the HDAC2-CREB -NR2B pathway.

Further supporting the role of HDAC2, CREB and NR2B in the learning and memory dysfunction of the offspring, we showed that SAHA (an HDAC inhibitor that mainly inhibits HDAC2) treatment 2 hours before each Morris water maze trial reversed the impaired learning and memory and the alterations in expression of HDAC2, CREB and NR2B mRNA and protein in the hippocampus. Many lines of evidence showed that HDAC inhibitors that mainly inhibit HDAC2 potentiate learning and memory. The class I HDAC inhibitor RGFP963 can enhance the consolidation of cued fear extinction [51]. Kinetically selective HDAC2 inhibitors rescued the memory deficits in mice with neurodegenerative disease by increasing H4K12 and H3K9 histone acetylation in hippocampal neurons [52]. SAHA facilitated fear extinction of rats by enhancing the expression of hippocampal NR2B-containing NMDA receptors. The levels of acetylated histones in the hippocampus increased significantly 2 hours after SAHA administration and were accompanied by enhanced binding of p-CREB to its binding site at the promoter of the NR2B gene [17], resulted in the increase of of NR2B mRNA levels, but not NR1 or NR2A mRNA. Therefore, we administered SAHA to the offspring 2 hours before every water maze trial. It is impossible to know whether the learning and memory improvements caused by SAHA in animals exposed to isoflurane are due to its general compensatory effects or a specific reversal of the effects of isoflurane. However, the effects of SAHA on animals exposed to isoflurane, along with the effects of isoflurane on HDAC, suggest that isoflurane may act on HDAC to affect learning and memory. The results of the present study provided the first demonstration that an HDAC inhibitor reverses the learning and memory impairments caused by maternal isoflurane exposure during late pregnancy. β-amyloid protein accumulation, caspase activation [53, 54], inositol 1,4,5-trisphosphate (IP3) receptor activation [36, 55] and calcium dysregulation [56] are critical pathological mechanisms in the neurotoxicity caused by isoflurane. It remains to be clarified whether these mechanisms are involved in the learning and memory impairment caused by maternal isoflurane exposure.

The maintenance of normal cognition is known to require precise excitatory–inhibitory (E/I) balance [57]. Disrupted NMDA-receptor signaling may be a molecular substrate common to a number of neurodevelopmental, neuropsychiatric disorders [58]. NR2B is an excitatory receptor and plays a critical role in the maintenance and formation of normal learning and memory. NR2B signaling can be maintained at a normal range to keep the brain in E/I balance. The rats in the CS group in the current study were normal offspring that had not exposed to isoflurane during pregnancy and had normal levels of inhibitory receptors. It is possible that they could maintain normal NR2B function by autoregulation or other pathways. NR2B protein expression in the I2, I4 and I8 groups had been inhibited by maternal isoflurane exposure. Therefore, the expression levels of NR2B protein in these rats could be increased by SAHA via the HDAC2-CREB-NR2B pathway [16, 17] to maintain the E/I balance. Protein levels are dependant on numerous factors, including gene transcription, translation and the number and functional state of cells that produce the protein. Maybe there are some unknow factors affecting the mRNA translating to protein, thus result in the increasing degree of NR2B protein was different from that of NR2B mRNA in the groups of I2S, I4S and I8S. Future studies are needed to determine if there are other factors affecting the translation of mRNA to protein in this pathway.

The present study only assessed the ultrastructural and molecular changes in the offspring brains after the behaviour test. The acute changes in the fetal brains immediately or several hours after isoflurane exposure had not been evaluated. Whether pretreatment with SAHA prior to isoflurane exposure will block all the harmful effects caused by isoflurane in offspring is not know yet. These questions require further clarification.

Taken together, the results in the present study suggested that maternal exposure of rats to isoflurane during late pregnancy impairs the spatial learning and memory of the offspring. This effect was associated with damage to hippocampal neurons, the overexpression of HDAC2 mRNA and the subsequent downregulation of CREB mRNA and NR2B. HDAC2 inhibition improved the impaired learning and memory of the offspring induced by maternal exposure to isoflurane.
